# Oral Health-Related Quality of Life in Breast Cancer Patients in the Northern Region of Saudi Arabia

**DOI:** 10.3390/healthcare11081189

**Published:** 2023-04-20

**Authors:** Zafar Ali Khan, Namdeo Prabhu, Rakhi Issrani, Amjad Abdulrahman S. Albulayhid, Shahad Mohammed Mlih Alruwaili, Rola Hliel Gadoe Alruwaili, Basant Mousa Alsiyat, Alzarea K. Bader, Mohammed Ghazi Sghaireen, Krishna Rao, Muhammed Nadeem Baig

**Affiliations:** 1 Department of Oral & Maxillofacial Surgery and Diagnostic Sciences, College of Dentistry, Jouf University, Sakaka 72388, Saudi Arabia; 2 Department of Preventive Dentistry, College of Dentistry, Jouf University, Sakaka 72388, Saudi Arabia; 3 College of Dentistry, Jouf University, Sakaka 72388, Saudi Arabia; 4 Department of Prosthetic Dental Sciences, College of Dentistry, Jouf University, Sakaka 72388, Saudi Arabia

**Keywords:** breast cancer, chemotherapy, oral health, quality of life

## Abstract

Background: Breast cancer is one of the most prevalent diseases, and ignoring dental health care before and after treatment can have severe long-term consequences. Additionally, this may have a negative impact on the patient’s general quality of life. Aim: The aim of the present study was to assess oral health-related quality of life (OHRQoL) among breast cancer patients and identify possible factors associated with the outcome. Methodology: In this observational cross-sectional study, 200 women who had received breast cancer therapy and were being followed up at a hospital made up the sample. The study was conducted between January 2021 and July 2022. Information on sociodemographic characteristics, general health, and breast cancer was recorded. The decayed, missing, and filled teeth index was used in clinical examinations to identify caries experience. OHRQoL was evaluated using the Oral Health Impact Profile (OHIP-14) questionnaire. After adjusting for confounding variables, a logistic regression analysis was used to determine the related factors. Results: The mean OHIP-14 score was 11.48 (SD 1.35). There was a 63.0% prevalence of negative impacts. Age and the time frame from cancer diagnosis were found to be significantly linked with the outcome by binary logistic regression analysis. Conclusions: Breast cancer survivors who were ≤55 years old and the time elapsed since diagnosis was less than 36 months had a poor OHRQoL. To lessen the negative impacts of cancer treatment and enhance quality of life, patients with breast cancer need special oral care and should be monitored before, during, and after cancer treatment.

## 1. Introduction

In 2017, there were 9.6 million cancer deaths and 24.5 million new cases of cancer worldwide [1]. The biggest cause of cancer-related death in women worldwide is breast cancer [2]. A shift in the population’s age composition, population expansion, and a rise in age-specific incidence rates may be the causes of the 35% increase in incident cases of breast cancer during the past few decades [1]. Despite a rise in incidence, there has been a 40% reduction in breast cancer death rates since their peak, most likely due to an adequate combination of early detection programs and advancements in adjuvant therapy [3].

Between 1990 and 2016, the prevalence of breast cancer in Saudi Arabia increased by up to 10 times [4,5]. This may be a result of the Saudi populace acquiring Western habits like smoking [6]. According to the Saudi government’s 2030 vision, it will be much easier for the health sector to prevent cancer by analyzing independent risk factors, improve health, and control cancer outcomes by treating cancer symptoms [7]. Due to a variety of national screening programs and early cancer diagnosis in new cases, the survival rate for cancer patients is increasing in Saudi Arabia [8]. Hence, cancer survivors require ongoing assessments and interventions in order to maintain quality of life and enhance health management and outcomes [9,10,11].

Many therapies are employed to treat the malignancy and promote disease-free survival [12]. Unfortunately, there are a lot of undesirable outcomes of these treatments that are harmful to health and lower quality of life [13,14]. Studies have shown that 40–90% of cancer patients who get chemotherapy may develop oral side effects [15,16,17]. The oral manifestations that cause the most discomfort, both immediately and over time, are xerostomia, mucositis, tooth loss, and gingival hemorrhage [18]. Additionally, it has been established that cancer patients’ decreased salivary flow increases their risk of oral infections and makes them more vulnerable to dental caries. These issues can also affect their ability to speak, chew, and swallow, which lowers their quality of life [19]. It has been discovered that there is less public awareness of breast cancer patients who have oral problems [20].

During breast cancer therapy, health-related quality of life has been used as a measure to evaluate perceptions and therapeutic results [21]. In order to fully understand the impact of oral problems on the lives of breast cancer patients, the measurement of oral health-related quality of life (OHRQoL), which is an addition to normative measurements, is helpful [22]. These actions provide patients with realistic expectations and may improve their adherence to treatment [21]. A number of evaluation instruments have been developed with the goal of evaluating OHRQoL. The Oral Health Impact Profile (OHIP-14) is one such assessment tool that has been widely used in other populations [23] and has been translated and validated for use in the Arabian population [24].

Studies in the local community help to manage patient awareness programs for people who have a higher risk of developing cancer [25,26]. Such knowledge can help with the establishment and use of prevention strategies as well as the rehabilitation and enhancement of these people’s quality of life. OHRQoL of breast cancer survivors has only been studied in a few studies carried out in Saudi Arabia [10,27], indicating that more study of this population is required. Additionally, no research has looked into how oral issues may affect the OHRQoL of breast cancer survivors in Saudi Arabia’s northern region. Thus, the aim of the present study was to evaluate the impact of oral problems on the OHRQoL in women undergoing breast cancer.

## 2. Methods and Materials

### 2.1. Study Design

A cross-sectional observational study was conducted using information gathered between January 2021 and July 2022. The Local Committee of Bioethics at Jouf University granted ethical permission for this study (approval number 4-08-42), and all the procedures used in it complied with the Helsinki Declaration.

### 2.2. Sample Calculation

A sample size calculator (found at http://www.raosoft.com/samplesize.html accessed on 22 November 2020) was used to determine the sample size based on the mean and standard deviation of OHRQoL values from the pilot study. The number of participants needed to detect a statistically significant difference of 10% in OHRQoL mean scores with 80% power and a 95% confidence level was 180. A sample size of 200 was chosen after adjusting for the 10% likelihood of incomplete responses.

### 2.3. Sample Population and Characteristics

The patients diagnosed with breast cancer (ICD-10, C50) at the King Abdulaziz Specialist Hospital, Al Jouf, Saudi Arabia, were involved. The King Abdulaziz Specialist Hospital provides general specialties in addition to being a local reference for oncological care.

### 2.4. Inclusion Criteria

(1) Saudi women aged 18 years or older;
(2) A confirmed case of breast cancer with a histopathology report;
(3) Records should be present within the database.

### 2.5. Exclusion Criteria

(1) Syndromic patients with orofacial anomalies that can affect oral conditions, e.g., Treacher Collins;
(2) History of trauma to the facial skeleton.

Patients who met the selection criteria were chosen as participants using a non-probability convenience sample. After being identified in the hospital’s database, the patients were recruited. The participation was voluntary, and written informed consent was obtained.

### 2.6. Data Collection and Survey Tool

#### 2.6.1. Structured Interview and Clinical Examination

A structured interview was conducted to gather data on the sociodemographic and lifestyle characteristics of the patient, such as age, education level, monthly income [28], marital status, and smoking. Additionally, data were gathered regarding the type of cancer, prior cancers, treatments used, time elapsed since cancer diagnosis, information on concomitant chronic diseases (such as diabetes) other than cancer, and self-reported depression. The data from the patient charts were used to supplement these findings.

The following oral health issues were also noted by the patients: oral pain, xerostomia (dry mouth), perceived tooth loss, decayed teeth, restored teeth, and the appearance of any oral lesions. Four interviewers who have completed training exercises conducted the interviews (1st, 2nd, 3rd, and 4th authors).

Using “universal precautions”, an oral examination was conducted by a single qualified and experienced examiner (1st author). The participants were assessed while seated in high-back chairs under white light from an LED torch. Dental diagnostic equipment and disposable wooden tongue retractors were used for the oral health examination. The caries experience was evaluated using the DMFT index [29]. In order to determine whether there were any lesions in the oral mucosa, Hipólito and Martins’ classification system was used [30]. The participant’s oral conditions were recorded on a pre-formed proforma.

#### 2.6.2. OHIP-14 Questionnaire

The 14-item OHIP is a socio-dental questionnaire that is inexpensive, easy to implement, and increases community access. In order to provide the best possible targeted treatment, it helps to identify the effects on people’s quality of life. Slade and Spencer created the OHIP and evaluated it to determine how oral health affects a person’s quality of life [23]. The conceptual model used by Locker to construct the initial version of OHIP made the assumption that the quality of life content associated with oral health exhibits seven conceptual dimensions: functional limitation, physical pain, psychological discomfort, physical disability, psychological disability, social disability, and social handicap [22]. It is available in several languages and is easily adapted to different cultures [31]. Results from the literature on the use and effectiveness of OHIP show that the instrument is sensitive enough to capture variations in the influence of oral conditions [32,33].

OHRQoL was assessed using the Arabic version of the OHIP-14 questionnaire, which has been validated for the Saudi population [24]. The OHIP-14 consists of 14 items (two items per domain) that are assigned to each of the seven domains of functional limitation, physical discomfort, psychological discomfort, physical disability, social impairment, and social handicap. The following statements are given a Likert scale score between 0 and 4: never, hardly ever, occasionally, often, and very often.

For the study and interpretation of the impacts identified using the OHIP-14, the prevalence and severity of impact were estimated as follows [34,35]:

1. The sum of all item scores—which ranged from 0 to 56 points—was used to calculate the severity (total score), with higher scores signifying a stronger negative impact felt by the respondent;
2. Based on a response of “often” or “very often” to at least one of the 14 items, the prevalence of impact was calculated. An answer of “often” or “very often” on at least one of the items was considered suggestive of a person “with impact”, and the domain scores were derived by adding the item scores.

### 2.7. Data Analysis

Data were analyzed using the Statistical Package for the Social Sciences, version 20.0 (IBM Corp., Armonk, NY, USA). The outcome was a negative impact on OHRQoL. The independent variables were age (defined by the mean ≤55/>55 years), marital status (married/not married), education (primary/secondary/bachelor/diploma/masters/PhD), income (SAR ≤10,000/>10,000), smoking (yes/no), self-reported depression (yes/no), diabetes (yes/no), previous cancer (yes/no), type of breast cancer (invasive duct carcinoma/other), time since diagnosis (≤36/>36 months), radiotherapy (yes/no), chemotherapy (yes/no), oral pain (yes/no), xerostomia (yes/no), tooth loss (yes/no), restored teeth (median ≤3/>3 teeth), decayed teeth (≤1/>1 tooth), and more than two oral lesions (yes/no). 

Using frequency tables, numbers, and percentages for each item on the study instrument, a descriptive analysis of the data was done. With the exception of age and educational level, where the Mann–Whitney U test was utilized, the association of dimensions of the influence of oral health and socio-demographic characteristics was tested by the independent *t* test. A logistic regression model was used to assess the factors linked to the detrimental effect on OHRQoL. Following initial univariate analysis, the binary logistic regression, and odds ratios (OR) were calculated for the independent variables with *p* values ≤ 0.25 [36].

## 3. Results

### 3.1. Baseline Characteristics of Participants

A total of 256 breast cancer patients were contacted, and 200 women who had survived the disease made up the final sample, yielding a response rate of 78.1%. The socio-demographic details and oral health issues in the sample under study are shown in Table 1. The majority of participants (*n* = 119; 59.5%) were under the age of 55, were married (*n* = 123; 61.5%), had completed their secondary education (*n* = 43; 21.5%), lived in households with an annual income of less than SAR 10,000 (*n* = 107; 53.5%), and did not smoke (*n* = 176; 88.0%). Among the participants, 56 (28.0%) said they had depression, while 69 (34.5%) said they had diabetes. According to the features of breast cancer, the majority of women (*n* = 184; 92.0%) had never had cancer before, had been diagnosed with invasive duct carcinoma (*n* = 141; 70.5%), and had been diagnosed for less than 36 months for 103 (51.5%) participants. None of these factors had a statistically significant relationship with the overall OHIP-14 score.

Regarding the examination of oral perceptions, 80 (40.0%) participants reported having dental pain, 116 (58.0%) reported having xerostomia, 65 (32.5%) reported having lost at least one tooth, 78 (39.0%) had more than one decayed tooth, 102 (51.0%) had more than three restored teeth, and 106 (53.0%) had more than two oral lesions. None of these factors had a statistically significant relationship with the overall OHIP-14 score (*p* > 0.05).

#### 3.1.1. Prevalence of OHIP-14 Impacts

The distribution of answers to the 14 OHIP items is shown in Table 2. Prevalence ranged from 5.0% to 20.0% using the percentage reporting impacts “often” or “very often”, with “problem pronouncing words”, “painful aching in mouth”, “uncomfortable to eat”, “self-conscious”, and “difficult to relax” being the most prevalent.

The prevalence of a negative impact and the mean OHIP-14 domain scores are shown in Table 3. Social handicap had the highest mean score (1.71 ± 0.45), which was followed by psychological discomfort (1.68 ± 0.46), social disability (1.67 ± 0.46), physical disability (1.66 ± 0.47), psychological disability (1.60 ± 0.49), functional restriction (1.59 ± 0.49), and physical pain (1.54 ± 0.49). Most responses were categorized as having “without impact” across all domains. The mean overall score was 11.48 (± 1.35), and 126 women (63.0%) had a negative impact on OHRQoL.

#### 3.1.2. Responses to the OHRQoL Ratings

The findings of the adjusted logistic regression analysis of the associations between the independent variables and the prevalence of impact are shown in Table 4. Age, depression, diabetes, breast cancer diagnosis, perceived tooth loss, and time since diagnosis were determined to have *p* values ≤ 0.25 in univariate analysis and were therefore added as independent variables to the binary logistic regression. According to this analysis, women under the age of 55 and those whose diagnosis occurred within the last 36 months had respectively 4.51-fold and 5.10-fold higher odds of having a negative influence on OHRQoL than women over the age of 55 and those whose diagnosis occurred within the past 36 months.

## 4. Discussion

OHRQoL and related characteristics were evaluated in the current study among breast cancer survivors. OHRQoL was negatively impacted by more than half of the women, and the regression analysis linked this to age and the amount of time after a breast cancer diagnosis. In a recent study, Jardim et al. found that xerostomia and the number of restored teeth were related to poor OHRQoL in more than half of the women (mean OHIP-14 score of 12.8) [37]. Breast cancer survivors had a mean OHIP-14 score of 18.6, which suggested a worse OHRQoL when compared to a control group of women without breast cancer, according to a longitudinal study by Taichman et al. [38]. The same study found that OHRQoL had a tendency to get better with time, showing people’s capacity for adaptation and providing an explanation for the lower mean score found in the current study (11.48), where the average time after diagnosis was 31.9 months.

The prevalence of 63.0% and mean OHIP-14 score of 11.48 points in the current study indicate that breast cancer survivors have lower OHRQoL than the general population. The prevalence of oral health problems reported by cancer patients was found to be much higher (86.1%) in the Saudi Arabian population, underlining the severity of this condition in Saudi Arabia [19]. Comparable results were also seen across different populations [37,39].

The majority of women reported no impact on any OHIP-14 domains. Nevertheless, the social disadvantage (1.71) and psychological discomfort (1.68) mean scores were the highest. The domains with the highest mean ratings in the study by Jardim et al. were psychological discomfort (2.78) and social disability (2.68) [37]. The physical pain domain in the study by Taichman et al. had the highest mean (3.03), followed by psychological discomfort (2.82) [38]. It is evident that oral issues significantly affect psychological features in breast cancer survivors, regardless of how long it has been since the diagnosis, given that the psychological discomfort domain in each of these investigations had very high means. The social handicap domain in the current study had the highest mean, which seemed to be linked to the esthetic and functional effects of oral issues in this sample.

The majority of individuals in this study experienced xerostomia, had more restored teeth (>3 teeth), and had more than two oral lesions. According to Jardim et al., xerostomia and having more restored teeth had a detrimental effect on OHRQoL in breast cancer survivors [37]. Oral difficulties are worse in breast cancer survivors, and these clinical traits appear to have an effect on OHRQoL [18]. Clinical oral features that typically have a detrimental effect on OHRQoL include dental caries, tooth loss, periodontal disease, and xerostomia [40]. Breast cancer survivors who experience xerostomia may be affected by hormonal changes, prior breast cancer therapy, medication use, and psychological variables, including stress and anxiety [37,38]. Only one study examined salivary flow in breast cancer survivors, reporting normal salivation and no association with the OHIP-14 score. This is despite the fact that xerostomia has been linked to worse OHRQoL in patients who have undergone head and neck radiotherapy, type II diabetes, and Sjogren’s syndrome [38]. In this study, a potential explanation for why the majority of participants had xerostomia could be linked to the fact that the majority of them (66.0%) had undergone chemotherapy, 34.5% had diabetes, and 28.0% had self-reported depression, all of which have the potential to result in xerostomia.

Less than half of the individuals in this study self-reported having depression. This is in line with the findings of Almutairi et al., who found that depression was present in 29.0% of Saudi cancer patients [30]. In contrast, cancer patients in Saudi Arabia had a significant proportion of depression, according to studies by Ahmed et al. [41] and Almigbal et al. [42]. The lack of research on self-reported psychological symptoms among cancer patients in Saudi Arabia was also addressed by these authors. Izci et al. state that depression is more prevalent among breast cancer survivors than in the general population and has been associated with a lower quality of life in terms of health [43]. The high prevalence of psychological symptoms may prompt oncologists to take psychological demands into account when managing and treating cancer.

Compared to women over 55, women under 55 had a 4.51-fold higher chance of having a negative effect on OHRQoL. The major risk factor for breast cancer is age. Up to the age of 50, incidence rates rise quickly; beyond that, they rise more gradually [44]. In this study, participants with less education had higher mean OHIP-14 scores than participants with master’s or doctoral degrees. This may be caused by the inadequate general health education that individuals receive throughout their academic careers. This result supports the assertions of Almutairi et al. that education has a beneficial impact on quality of life, despite the fact that their study focused solely on colorectal cancer [30]. This problem, in our opinion, can be remedied by giving this group of patients adequate time with the specialists to enable attentive listening, thorough explanations of the nature of the disease, and encouragement of any inquiries.

Participants with a monthly household income of less than SAR 10,000 exhibited a considerably higher mean difference in OHIP-14 scores than those with a monthly household income of more than SAR 10,000. Lower income levels have been linked to higher mean OHIP-14 scores, according to earlier studies [45,46]. The results of this study are in line with the literature evidence linking poor dental health to overall population problems [19]. Low-income patients struggle to afford dental care, and oncologists struggle to educate them adequately about oral care, which makes the issue worse [19]. As a result, healthcare practitioners should emphasize to cancer patients who are living in poverty the benefits of basic oral care and recommend or refer them to expert dental consultation. It is crucial to remember that the development of breast cancer is also connected to the urbanization of society, despite the fact that there is evidence to suggest that women from higher socioeconomic classes are at a higher risk [44]. In this study, the mean difference in OHIP-14 scores between smokers and non-smokers was considerably larger for smokers. Our findings were in line with earlier research that showed smoking has a detrimental effect on dental health [47].

Almost 20% of cancer deaths globally are a result of smoking [48]. Saudi Arabia ranks fourth in the world for tobacco usage; men are thought to smoke about 26.5% of the time, compared to women, who smoke 9% of the time [49,50]. Hence, smoking cessation programs for cancer patients must be vigorously pursued.

Our research showed that, in order to limit the probability of poor oral health having a negative impact on quality of life, standard cancer therapy procedures must include oral health management. Additionally, this study will advance knowledge of the state of oral health among breast cancer survivors in Saudi Arabia’s northern region.

### Limitations

The limits of the current investigation must be taken into consideration. First, a cross-sectional design might not be able to establish causality. However, given the dearth of literature in this area, our findings may serve as a guide for future study by assisting in the formulation of hypotheses and by providing crucial information to target susceptible individuals. The sample size being so small is still another drawback. To corroborate our findings, additional multicenter, large-sample studies with a longer time frame are suggested. This study was not based on clinical evaluation, which would have overestimated the prevalence of these findings, but rather on self-reports of subjective oral health status (such as xerostomia) and psychosocial problems. Additionally, the fact that our study was only conducted in a single Saudi Arabian city hospital limits its capacity to be generalized, but given the shared cultural background of the Saudi people, we would anticipate that the results would still be applicable to the broader population. Lastly, given that there was no control group in this study, the suggestions should be carefully considered. It is recommended to conduct longitudinal studies with other demographics and a carefully chosen control group in order to confirm and better understand the outcomes of the current study.

## 5. Conclusions

The OHRQoL of breast cancer survivors in the current study was significantly negatively impacted, and this effect was correlated with age and the amount of time after breast cancer diagnosis. Cancer patient care practices should involve oral health evaluation and treatment in order to maintain proper oral hygiene. The dentist must be a member of the multi-professional team due to the significance of early detection of oral problems. This will significantly improve the quality of life for cancer patients.

## Figures and Tables

**Table 1:** Associations between characteristics of women with breast cancer and OHIP-14 scores.

Variables	*N* (%)	Mean (± SD)	*p*-Value
Age (in years)			0.754
≤55	119 (59.5)	31.95 (5.25)	
>55	81 (40.5)	31.70 (6.11)	
Marital status			0.074
Married	123 (61.5)	32.41 (5.87)	
Not married	77 (38.5)	30.96 (5.05)	
Education *			0.77
Primary	57 (28.5)	32.28 (5.35)	
Secondary	43 (21.5)	31.51 (6.11)	
Bachelor	40 (20.0)	32.07 (5.90)	
Diploma	21 (10.5)	32.85 (6.32)	
Masters	23 (11.5)	30.65 (4.94)	
PhD	16 (8.0)	31.12 (4.44)	
Household income (in SAR)			0.503
≤10,000	107 (53.5)	31.96 (6.18)	
>10,000	93 (46.5)	31.73 (4.88)	
Smoking habit			0.253
Smoker	24 (12.0)	33.08 (6.35)	
Non-smoker	176 (88.0)	31.68 (5.49)	
Depression			0.260
Yes	56 (28.0)	32.57 (6.29)	
No	144 (72.0)	31.57 (5.30)	
Diabetes			0.615
Yes	69 (34.5)	32.13 (6.04)	
No	131 (65.5)	31.70 (5.37)	
Previous cancer			0.168
Yes	16 (8.0)	30.00 (5.31)	
No	184 (92.0)	32.01 (5.61)	
Diagnosis of breast cancer	141 (70.5)	31.59 (5.54)	0.313
Invasive duct carcinoma Other	59 (29.5)	32.47 (5.74)	
Time since diagnosis			0.301
≤36 months	103 (51.5)	31.45 (4.98)	
>36 months	97 (48.5)	32.27 (6.18)	
Radiotherapy			0.767
Yes	129 (64.5)	31.76 (5.71)	
No	71 (35.5)	32.01 (5.43)	
Chemotherapy			0.152
Yes	132 (66.0)	31.44 (5.36)	
No	68 (34.0)	32.64 (6.00)	
Oral pain			0.285
Yes	80 (40.0)	32.37 (6.02)	
No	120 (60.0)	31.50 (5.29)	
Xerostomia			0.442
Yes	116 (58.0)	31.59 (5.86)	
No	84 (42.0)	32.21 (5.22)	
Perceived tooth loss			0.821
Yes	65 (32.5)	31.98 (5.11)	
No	135 (67.5)	31.79 (5.84)	
Decayed teeth			0.637
≤1	122 (61.0)	31.70 (5.89)	
>1	78 (39.0)	32.08 (5.14)	
Restored teeth			0.462
≤3	98 (49.0)	32.15 (5.19)	
>3	102 (51.0)	31.56 (5.97)	
≥2 oral lesions			0.824
Yes	106 (53.0)	31.38 (5.59)	
No	94 (47.0)	32.38 (5.59)	

* Mann–Whitney U test.

**Table 2:** Distribution of responses to the 14 OHIP items.

OHIP Domain	OHIP Items	Never *N* (%)	Hardly Ever *N* (%)	Occasionally *N* (%)	Often *N* (%)	Very Often *N* (%)
Functional Limitation	Trouble pronouncing words	60 (30.0)	60 (30.0)	29 (14.5)	21 (10.5)	30 (15.0)
	Felt sense of taste worsened	60 (30.0)	54 (27.0)	45 (22.5)	26 (13.0)	15 (7.5)
Physical Pain	Experiencing painful aching in the mouth	63 (31.5)	44 (22.0)	37 (18.5)	25 (12.5)	31 (15.5)
	Uncomfortable to eat foods	73 (36.5)	39 (19.5)	33 (16.5)	27 (13.5)	28 (14.0)
Psychological Discomfort	Felt self-conscious	63 (31.5)	66 (33.0)	22 (11.0)	30 (15.0)	19 (9.5)
	Felt tense	79 (39.5)	59 (29.5)	38 (19.0)	13 (6.5)	11 (5.5)
Physical Disability	Diet has been unsatisfactory	94 (47.0)	50 (25.0)	20 (10.0)	12 (6.0)	24 (12.0)
	Had to interrupt meals	84 (42.0)	62 (31.0)	20 (10.0)	22 (11.0)	12 (6.0)
Psychological Disability	Difficult to relax	77 (38.5)	52 (26.0)	21 (10.5)	10 (5.0)	40 (20.0)
	Been a bit embarrassed	82 (41.0)	59 (29.5)	25 (12.5)	19 (9.5)	15 (7.5)
Social Disability	Irritable with other people	85 (42.5)	48 (24.0)	27 (13.5)	27 (13.5)	13 (6.5)
	Difficulty doing usual jobs	78 (39.0)	49 (24.5)	40 (20.0)	18 (9.0)	15 (7.5)
Social Handicap	Felt less satisfying life	87 (43.5)	63 (31.5)	23 (11.5)	12 (6.0)	15 (7.5)
	Totally unable to function	70 (35.0)	59 (29.5)	37 (18.5)	21 (10.5)	13 (6.5)

**Table 3:** Mean OHIP-14 domain scores and prevalence of impact.

OHIP-14	Mean (±SD)	Domain Min–Max	With Impact *N* (%)	Without Impact *N* (%)
Functional limitation	1.59 (0.49)	2.0–10.0	81 (40.5)	119 (59.5)
Physical pain	1.54 (0.49)	2.0–10.0	91 (45.5)	109 (54.5)
Psychological discomfort	1.68 (0.46)	2.0–10.0	63 (31.5)	137 (68.5)
Physical disability	1.66 (0.47)	2.0–10.0	67 (33.5)	133 (66.5)
Psychological disability	1.60 (0.49)	2.0–10.0	80 (40.0)	120 (60.0)
Social disability	1.67 (0.46)	2.0–10.0	65 (32.5)	135 (67.5)
Social handicap	1.71 (0.45)	2.0–10.0	57 (28.5)	143 (71.5)
Total score	11.48 (1.35)	7.0–51.0	126 (63.0)	74 (37.0)

**Table 4:** Binary logistic regression analysis between the prevalence of impact and independent variables.

	*N* (%)	With Impact *N* (%)	OR (Adjusted)	95% C.I.		*p*-Value
				Lower	Upper	
Age (in years)			4.51	1.025	19.796	0.046 *
≤55	119 (59.5)	116 (61.1)				
>55	81 (40.5)	74 (38.9)				
Depression			4.44	0.495	39.825	0.183
Yes	56 (28.0)	55 (28.9)				
No	144 (72.0)	135 (71.1)				
Diabetes			0.32	0.078	1.284	0.107
Yes	69 (34.5)	63 (33.2)				
No	131 (65.5)	127 (66.8)				
Diagnosis of breast cancer			1.99	0.493	8.106	0.332
Invasive duct	141 (70.5)	136 (71.6)				
carcinoma						
Other	59 (29.5)	54 (28.4)				
Time since diagnosis			5.10	0.978	26.578	0.053 *
≤36 months	103 (51.5)	101 (53.2)				
>36 months	97 (48.5)	89 (46.8)				
Perceived tooth loss			3.76	0.442	31.958	0.225
Yes	65 (32.5)	64 (33.7)				
No	135 (67.5)	126 (66.3)				

* Statistically significant.

## Data Availability

The data set used in the current study will be made available on request from Rakhi Issrani at dr.rakhi.issrani@jodent.org.
